# Supplementary figures and images for: Type 1 Diabetes: an Association Between Autoimmunity, the Dynamics of Gut Amyloid-producing E. coli and Their Phages
Source: Sci Rep. 2019 Jul 4;9:9685. doi: 10.1038/s41598-019-46087-x (PMC6609616; doi:10.1038/s41598-019-46087-x)

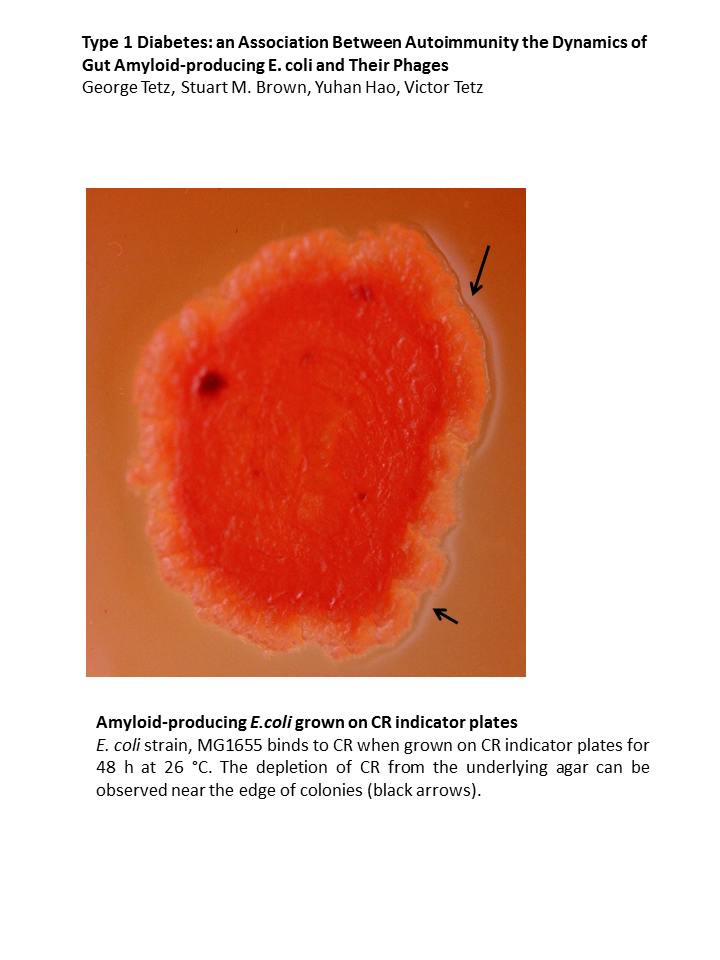

Supplement: Supplementary file 1 — Supplementary Figure S1 [file 41598_2019_46087_MOESM1_ESM.tif]
