# Supplementary material for: Type 1 Diabetes: an Association Between Autoimmunity, the Dynamics of Gut Amyloid-producing E. coli and Their Phages
Source: Sci Rep. 2019 Jul 4;9:9685. doi: 10.1038/s41598-019-46087-x (PMC6609616; doi:10.1038/s41598-019-46087-x)
Supplement: Supplementary file 4 — Supplementary Table S3 [file 41598_2019_46087_MOESM4_ESM.docx]

**Type 1 Diabetes: an Association Between Autoimmunity the Dynamics of Gut Amyloid-producing E. coli and Their Phages**

George Tetz, Stuart M. Brown, Yuhan Hao, Victor Tetz

Supplementary table 3

**P-value for *E. coli* Abundance Over Time**

| Control | | |
| --- | --- | --- |
| Time bin 1 | Time bin 2 | p-value |
| T0-300 | T300-600 | 0,489471 |
| T0-300 | T600-900 | 0,798039 |
| T0-300 | T900-1300 | 0,77033 |
|  |  |  |
| Seroconverted | | |
| T0-300 | T300-600 | 0,006304 |
| T0-300 | T600-900 | 0,003135 |
| T0-300 | T900-1300 | 0,03577 |
|  |  |  |
| T1D | | |
| T0-300 | T300-600 | 0,022669 |
| T0-300 | T600-900 | 0,021403 |
| T0-300 | T900-1300 | 0,011644 |
