# Supplementary material for: Type 1 Diabetes: an Association Between Autoimmunity, the Dynamics of Gut Amyloid-producing E. coli and Their Phages
Source: Sci Rep. 2019 Jul 4;9:9685. doi: 10.1038/s41598-019-46087-x (PMC6609616; doi:10.1038/s41598-019-46087-x)
Supplement: Supplementary file 5 — Supplementary Table S4 [file 41598_2019_46087_MOESM5_ESM.docx]

**Type 1 Diabetes: an Association Between Autoimmunity the Dynamics of Gut Amyloid-producing E. coli and Their Phages George Tetz, Stuart M. Brown, Yuhan Hao, Victor Tetz**

**Supplementary table 4**

**Association of disappearance of *E. coli* and development of seroconversion and/or T1D**

| **Patient #** | **Case** | **Age of seroconversion (days)** |
| --- | --- | --- |
| T025418 | T1D | 540 |
| E010937 | T1D | 905 |
| E006574 | T1D | 533 |
| E003251 | T1D | 358 |
| E003989 | seroconverter | 347 |
| E010629 | seroconverter | 945 |
| E018113 | seroconverte | 588 |
| E022137 | seroconverter | 562 |
| E026079 | seroconverter | 580 |
| T013815 | seroconverter | 350 |
| E001463 | control | N/A |
| E006547 | control | N/A |
| E006673 | control | N/A |
| E010590 | control | N/A |
| E018268 | control | N/A |
| E022852 | control | N/A |
| T014292 | control | N/A |
| E016924 | control | N/A |
